# Dicotyledon Weed Quantification Algorithm for Selective Herbicide Application in Maize Crops

**DOI:** 10.3390/s16111848

**Published:** 2016-11-04

**Authors:** Morten Stigaard Laursen, Rasmus Nyholm Jørgensen, Henrik Skov Midtiby, Kjeld Jensen, Martin Peter Christiansen, Thomas Mosgaard Giselsson, Anders Krogh Mortensen, Peter Kryger Jensen

**Affiliations:** 1 Department of Engineering, Aarhus University, 8000 Aarhus, Denmark; rnj@eng.au.dk; 2 The Maersk Mc-Kinney Moller Institute, University of Southern Denmark, 5230 Odense, Denmark; hemi@mmmi.sdu.dk (H.S.M.); kjen@mmmi.sdu.dk (K.J.); 3 AGROINTELLI, 8200 Aarhus, Denmark; mpc@agrointelli.com; 4 Danish Technological Institute, Robot Technology, 5230 Odense, Denmark; tmg@teknologisk.dk; 5 Department of Agroecology—Crop Health, Aarhus University, 4200 Slagelse, Denmark; anmo@agro.au.dk (A.K.M.); pkj@agro.au.dk (P.K.J.)

**Keywords:** weed crop discrimination, grid sprayer, herbicide reduction, site specific, sprayer boom, monocot and dicot coverage ratio vision (MoDiCoVi)

## Abstract

The stricter legislation within the European Union for the regulation of herbicides that are prone to leaching causes a greater economic burden on the agricultural industry through taxation. Owing to the increased economic burden, research in reducing herbicide usage has been prompted. High-resolution images from digital cameras support the studying of plant characteristics. These images can also be utilized to analyze shape and texture characteristics for weed identification. Instead of detecting weed patches, weed density can be estimated at a sub-patch level, through which even the identification of a single plant is possible. The aim of this study is to adapt the monocot and dicot coverage ratio vision (MoDiCoVi) algorithm to estimate dicotyledon leaf cover, perform grid spraying in real time, and present initial results in terms of potential herbicide savings in maize. The authors designed and executed an automated, large-scale field trial supported by the Armadillo autonomous tool carrier robot. The field trial consisted of 299 maize plots. Half of the plots (parcels) were planned with additional seeded weeds; the other half were planned with naturally occurring weeds. The in-situ evaluation showed that, compared to conventional broadcast spraying, the proposed method can reduce herbicide usage by 65% without measurable loss in biological effect.

## 1. Introduction

The general practice of controlling emerged weeds in arable fields consists of the uniform application of a selective herbicide. This ensures a competitive advantage for the crop without severely damaging it. However, the extensive use of herbicides in the last few decades [1] has been challenged by consumers as well as policy makers because of growing concerns regarding their potential environmental impact on drinking water reservoirs, fauna in watercourses, etc. [2]. In 2010, pesticide residues were found in 44% of the drinking water monitoring points in Denmark, and the accepted limit of 0.1 μg/L was exceeded in 15% of the monitoring points [3]. This led to stricter regulation of herbicides prone to leaching and greater cost for the agricultural industry through taxation. These developments have encouraged investment in research on technologies that can reduce the amount of herbicides used in crops without affecting the herbicidal effect.

### 1.1. Research in Weed Infestation

Early research in this area used mean weed infestation levels as a measure of weed control needs. In [4,5], weeds occur in patches, which has a substantial impact on yield loss estimations. Indeed, this discovery formed the foundation of many attempts to reduce herbicide use and suggests that uniform or broadcast spraying is suboptimal. The mobility of weed patches over time was discussed by [6]. They concluded that infrequent samples on a large grid might result in maps that do not reflect the true spatial variation. They showed that it is difficult to formulate a general rule regarding optimal weed mapping frequency in part on account of the relevant species and seasonal dependencies. These studies support the need for real-time weed identification. Their conclusions were based on both their own experimental data as well as a literature review. Research by [7,8] suggest that several important weed species occur in patches, and these patches are relatively static for some species from one year to the next. The size of the patches may vary greatly, thereby resulting in a low correlation between weed infestation levels and locations.

### 1.2. Sprayer Systems

Currently used agricultural sprayers are built to uniformly apply herbicides across fields. However, new machinery with variable rate application technology has recently been developed, such as Amaspot from Amazon, which is based on the Weed-IT sensor developed by Rometron. Some scientific equipment [9] is now being marketed by Garford as well. These machines are a prerequisite for any type of herbicide-based site-specific weed control. When machinery for the variable application of herbicides is available, the remaining task is to control this equipment such that the necessary weed control is achieved.

Several tasks must be addressed to produce control signals for a sprayer system in this scenario. The first is to sense the weeds in the fields [10]. Data analysis is then needed to discriminate between crop and weed areas. The next step is to decide if weed control is needed by taking into account several issues, including the competitiveness of the crop and economic factors. The decision function will be a tradeoff between weed control and yield loss, which has been a topic of interest in risk management and modeling, as shown in [5,11].

### 1.3. Sensor Systems for Weed Detection

Several sensor-based approaches have been investigated in the past few years. With the aim of producing weed infestation maps, a remote sensing approach was used with some success [7,10,12]. Remote sensing relies on hyperspectral or multispectral images of fields that enable the detection of weed patches, given that the patches have a minimum size and density. Remote sensing for weed control suffers from drawbacks that render it a sub-optimal approach compared with other approaches. However, with increasing sensor resolution and cheaper equipment, this technology may experience a revival in the future, especially with the emergence of autonomous aerial systems (UAS). However, at present, ground-based sensing equipment appears more promising [10].

For ground-based sensing, high-resolution image data is the prevailing approach taken by researchers. The sensor type varies from off-the-shelf RGB cameras to specialized spectral cameras that utilize the general absorption of light of wavelengths in the vicinity of 670 nm and reflection of approximately 800 nm for vegetation. High-resolution images enable the use of shape and texture characteristics in addition to spectral characteristics. Instead of the detection of weed patches, local weed cover or density can be estimated at a sub-patch level, and single plant identification has been made possible [10].

To the best of the authors’ knowledge, no existing commercial system offers herbicide application on a single-plant basis, with the exception of systems for spot spraying large plants, such as volunteer potatoes in sugar beets [13,14,15] investigated the precision of a micro-spraying (or droplet-on-demand) indoor system with which they attained sub-centimeter precision with their sprayer. They conducted tests in which a computer vision system was used to detect black circles on a piece of polyvinyl chloride (PVC) while a wheel encoder estimated travel speed, thereby enabling timing calculations of the required delays between sensing and reacting. They concluded that, under indoor conditions, assuming that weed seedlings have sizes comparable with those of the black circles used in the test setup, the application of glyphosate can be reduced to 1/100 of the recommended amount for broadcast spraying. Similar results have been obtained in the case of maize under controlled conditions [16].

### 1.4. Plant/Weed Detection Systems

Handling the presence of crop plants means that the vegetation is a mixture of the crop and weeds. Several researchers have reported on systems that can distinguish crops from weeds with certain limitations [7,10,12,17,18,19]. These approaches disregard the presence of monocot weeds; however, by assessing the amount of dicot weeds, these approaches have been shown to provide an acceptable estimate for general weed cover. Several researchers have reported remarkable herbicide savings using this approach, and implementing such systems in conventional farming will be a huge step towards a more sustainable agricultural industry [10,20]. A recurring problem in data processing algorithms is caused by the difficulties in handling overlapping plants [7,21]. This circumstance introduces noise in the analysis and ultimately results in sub-optimal weed control. The authors of [22] addressed this by using a Monocot and Dicot Coverage Ratio Vision (MoDiCoVi) algorithm. This algorithm can estimate the ratio of dicotyledon pixels to monocot pixels, which is representative of the ratio of weeds to crop plants in products, such as cereal. This approach is based on the assumption that the relationship between the areas occupied by crops and weeds, as well as the yield loss, can provide better prediction than a relationship based on weed density [23].

### 1.5. Contribution and Structure of This Work

In past work [24], the authors designed and executed a large-scale, automated field trial supported by the Armadillo autonomous tool carrier robot. The Armadillo was equipped with a grid-spraying implement with artificial illumination, a two-camera sensor system, and six separately controlled sprayer nozzles. This corresponded to level 2 weed control (level 1 consists of separate plant treatment, while level 4 constitutes uniform treatment of the entire field) [10]. The scientific contribution of this work is an improvement in and a performance evaluation of the MoDiCoVi algorithm in situ. The algorithm is relatively complex, and thus a secondary aim of this work is to present the principles of operation of the proposed method.

This paper is divided into the following sections: (1) MoDiCoVi Dicotyledon weed quantifying algorithm briefly shows the principles of operation of the proposed method and modifications to the algorithm; (2) Evaluating experimental layout describes the in-situ plot trial that enables an efficient evaluation of the potential herbicide savings of the two algorithms; (3) Automated trial execution explains the technical details of the Armadillo robot and the grid-spraying tool. This section also describes the planning and control of the robot’s movement in an experiment; (4) Data pre-processing and visualization explains the data processing, filtering, and validation involved. Furthermore, the necessary visualizations that are needed to motivate the initial idea of potential herbicide savings through the two algorithms are described.

## 2. Materials and Methods

### 2.1. Image Acquisition and Segmentation

Images were acquired with a Basler Ace aca2000-50gc RGB camera (Basler AG, Ahrensburg, Germany) equipped with a Bayer patterned color filter in front of a complementary metal–oxide–semiconductor (CMOS) sensor. An excess green index was computed for a pixel in the raw image by observing a 2 × 2 square placed above the pixel such that the center of the pixel coincided with the lower-right corner of the square. The square always contained two green pixels, $G_{1}$ and $G_{2}$, a red pixel, *R*, and a blue pixel, *B*. Based on work by [25], but derived directly from the Bayer pattern, the normalized excess green color index was then computed as

(1) $$ExG=\frac{G_{1}+G_{2}-R-B}{R+G_{1}/2+G_{2}/2+B}$$

At this stage, the image was segmented using a fixed threshold; further, a binary vegetation/soil image was obtained. Pixels with values above the threshold were considered vegetation, whereas the other pixels were marked as soil. Examples of the images from the segmentation process can be seen in Appendix A.

### 2.2. MoDiCoVi Dicotyledon Weed Quantifying Algorithm

The proposed approach to quantifying dicotyledon weed plants in a monocotyledon crop such as maize is based on a close analysis of the contours of vegetation regions [26]. By observing vegetation contours instead of plants, the problem of overlapping plants can be mitigated to some extent. The MoDiCoVi algorithm consists of the following basic steps [22]:

1. The Bayer pattern is segmented into a binary vegetation/soil image.
2. Contours are located, and a subset of edge segments along the contours is chosen for further analysis.
3. The relative locations of nearby edge segments are calculated.
4. The probability distribution of coordinates of the nearby edge segments is calculated. Using this information, the number of weed pixels in the image is estimated.

**Figure 1:**
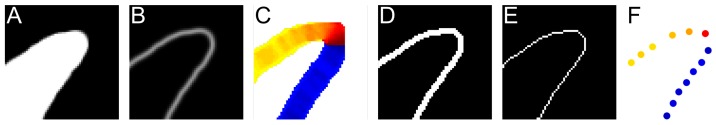
(**A**) Segmented leaf; (**B**) Edge magnitude response; (**C**) Edge phase response; (**D**) Thresholded edge magnitude response; (**E**) Thinned thresholded edge magnitude response; (**F**) Subdivision of edge phase responses.

Furthermore, by analyzing edge segment positions relative to other edge segments, the algorithm is not affected by changes in the position and orientation of the analyzed objects. In the following sub-sections, the components of the algorithm are described in detail. The image processing steps are shown in Figure 1.

#### 2.2.1. Find Directional Edges

From the binary image of the vegetation, the outline of the vegetation regions is located using an edge detection kernel. To obtain oriented edge segments, complex moments around every pixel in the input image are calculated using the following Equation [27]:

(2) $$I_{m,n}=\int_{-\infty }^{\infty }\int_{-\infty }^{\infty }{\left(x+iy\right)}^{m}{\left(x-iy\right)}^{n}\rho \left(x,y\right)dxdy$$

In this equation, $\rho \left(x,y\right)$ represents a window of the input intensity image multiplied by a Gaussian weighting function $\frac{1}{2\pi \sigma^{2}}\exp\left(\frac{-x^{2}-y^{2}}{2\sigma^{2}}\right)$, where *σ* is the bandwidth, and *x* and *y* describe the position relative to the windows’s center. The parameters *m* and *n* determine the shape to be searched for, and are set to 1 and 0, respectively, in order to locate edges. The computation was implemented as a convolution with a complex kernel. The result of the convolution with the edge detection kernel is an image where pixel values are represented by complex numbers. This is converted to a magnitude and phase response (see examples b and c in Figure 1) by converting the complex numbers to polar coordinates. To locate edges that are one-pixel wide, the magnitude image is first thresholded and then thinned until convergence. This yields the location of the edge segments. The associated orientation is determined from the phase response at the same location. To limit the amount of calculation required in the next step (calculation of relative edges), the original image is divided into 8 × 8 pixel squares, and only one edge segment is retained from each square. The edge segments are then stored in a list using their absolute positions (*x* and *y*) and orientations (*θ*). In the following, the *n*-th edge segment is denoted by $P_{n}$:

(3) $$P_{n}=\left[x_{n},y_{n}\theta_{n}\right]$$

#### 2.2.2. Calculating Relative Coordinates of Edge Segments

**Figure 2:**
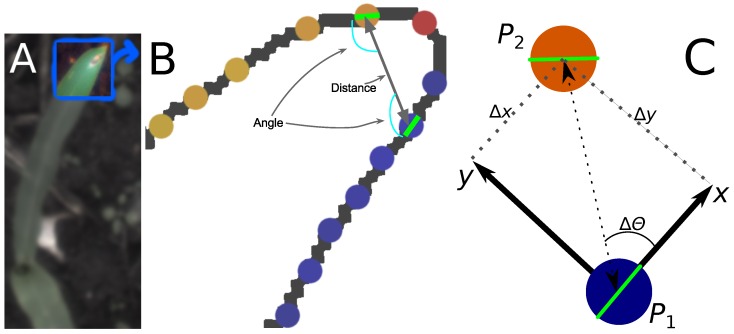
(**A**) Image of the leaf tip used in the example; (**B**) Edge segments along the leaf edge; (**C**) Two edge segments outlined with the relative coordinates from $P_{1}$ to $P_{2}$.

Given two edge segments $P_{1}$ and $P_{2}$, it is possible to describe the location of $P_{2}$ relative to $P_{1}$ by placing a new coordinate system centered on and aligned with $P_{1}$. This relative placement is denoted by $\Delta P_{21}$ and is described by the values Δy and Δθ, as shown in Figure 2. Only edge segment pairs separated by fewer than 125 pixels are considered. This threshold value is chosen such that the same plant is adequately visible from the investigated edge element, which supports high threshold values, while the number of edge segment pairs is kept at a manageable level (which supports low threshold values).

#### 2.2.3. Coordinate Distribution Analysis

**Figure 3:**
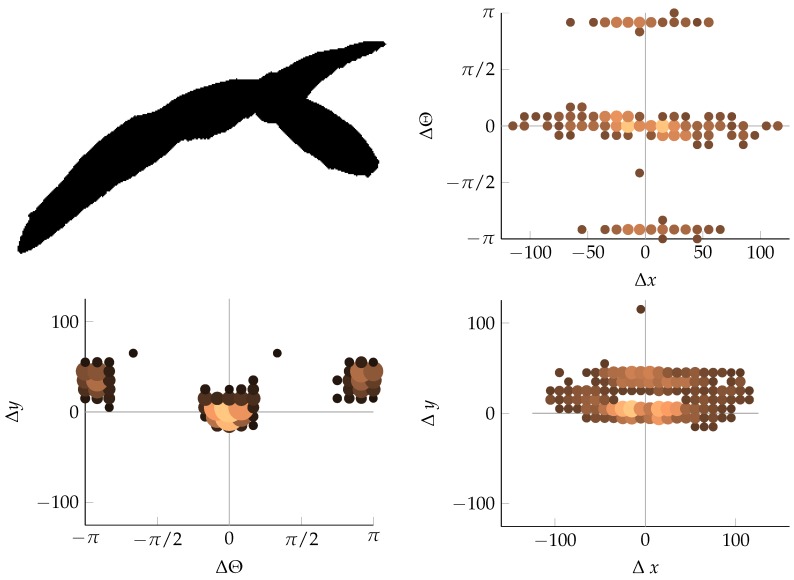
Monocots in 2D projection. The size and color of each dot denotes the number of occurrences of relative edge segments at the relevant position.

**Figure 4:**
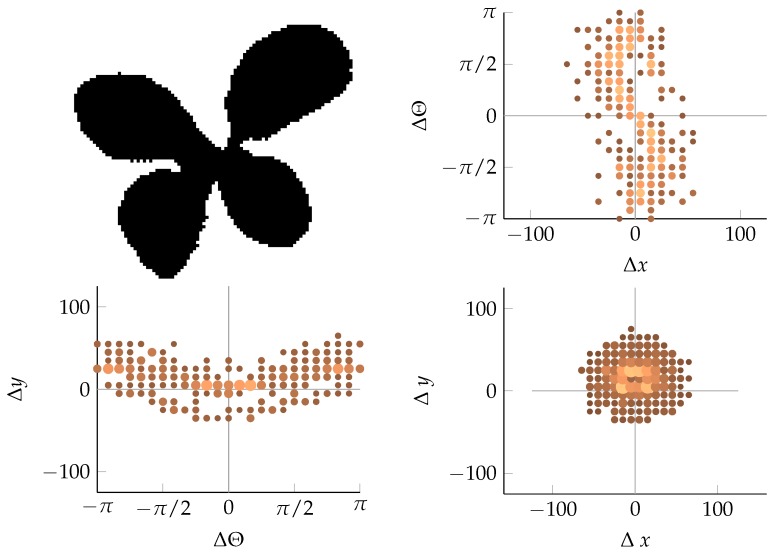
Dicots in 2D projections, which are similar to the representations in Figure 3.

When the relative coordinates of the relevant edge segment pairs have been determined, their distribution is investigated. By projecting the density of the relative coordinates onto a two-dimensional (2D) surface spanned by a combination of two of the relative coordinates, a fingerprint of the edge structure is generated. This type of fingerprint is interesting because monocots and dicots appear very distinctly in it. This is shown in Figure 3 and Figure 4. In Figure 3, the long, straight edges of monocots appear as distinct horizontal lines in the projection onto Δx and Δθ, whereas in Figure 4, the segments are more evenly spread across different angles, and the distance decreases as the weed leaves become smaller. More examples of different geometric shapes as well as more examples of vegetation can be seen in Appendix B. In addition to the relative coordinates (Δx, Δy, and Δθ), two extra features are included in the description of each pair of edge segments. These are the distance *d* between the edge segments and a measure, *k*, of the curvature of the path through the two edge segments. The features are derived from the raw relative coordinates as follows:

(4) $$\begin{array}{cc} d & =\sqrt{\Delta x^{2}+\Delta y^{2}} \end{array}$$

(5) $$\begin{array}{cc} k & =\frac{2-2cos(\Delta \theta )}{d} \end{array}$$

In order to estimate the distribution of the relative points, a framework inspired by Gaussian mixture models is applied. The concept is to measure the density of data near specific interest points. Nearness is determined by applying a Gaussian weight. The farther a point is from the point of interest, the smaller its weight. The density near these interest points is calculated using the following formula:

(6) $$G_{m}=\frac{e^{-\sum {\left[\frac{\Delta P-\vec{F_{cntr}}}{\vec{F_{\sigma }}}\right]}^{2}}}{N}$$

where $G_{m}$ is the density measured at Gaussian feature *m*, ΔP is the point difference, $F_{cntr}$ is the center of the Gaussian feature, $F_{\sigma }$ is the bandwidth of the feature, and *N* is the total number of relative points. The estimation of the weed area ratio is then performed through a linear combination of the Gaussian features through a linear combination as

(7) $$est=G_{1}\cdot w_{1}+G_{2}\cdot w_{2}+G_{3}\cdot w_{3}+\cdots +G_{m}\cdot w_{m}$$

where $w_{m}$ is the weight of the Gaussian feature *m* and est is the estimation. In order to estimate the weights, Gaussian feature positions, bandwidths, and a training set are required. An image can be considered as consisting of a random number of weeds and maize at random positions in the image. To reduce the dimensionality of the set to be annotated, the authors chose to annotate only individual plants instead of entire images, and allow the computer to randomly chose the manner of combining data from sets of plants into images. The training set is then reduced to the natural variation in the plants rather than in the relevant scene. An added benefit is that this approach adequately handles the case of overlapping plants, which can otherwise be difficult to annotate. When combined artificially in this manner, the exact extent of overlap is known. Using the artificially expanded dataset, a set of 450 random feature vectors is created. Each feature is created as defined in Equation (6) using a random location Fcntr sampled from a uniform distribution and a random bandwidth $F_{sigma}$ sampled from a log distribution. From this feature set, the authors seek to find the combination of 20 features that best describe the number of weeds in the dataset. This is accomplished by initializing with a random set of 20 features on which a linear regression model is created. The mean square error from this model is then used as the initial estimation error. Based on this error, a random feature that is part of the active set is exchanged for one outside the set. The error is then reevaluated. If the error is lower than the previously evaluated error, it becomes the new reference model. This search is repeated for *n* iterations. The regression model resulting from this process is the finished model.

#### 2.2.4. Adaptation to MoDiCoVi: Predicting the Amount of Weed Per Unit Area

In the previous version of the MoDiCoVi algorithm [22] the density feature (Equation (6)) was normalized by the number of total points to measure the ratio directly. However, this version of MoDiCoVi algorithm requires all points to be part of the model and not just the points which is described by the current Gaussian and is therefore overly sensitive to points not sufficiently described by the model. If, however, the definition of the Gaussian features are modified to do away with the normalization, each Gaussian must only represent the feature which it is selected to describe. As the area can be considered constant, i.e., the photographed area does not change, this can be regarded as a fixed value compared to the estimated ratio described earlier. Therefore, the normalization of the Gaussian feature is removed such that it becomes

(8) $$G_{m}=e^{-\sum {\left[\frac{\Delta P-\vec{F_{cntr}}}{\vec{F_{\sigma }}}\right]}^{2}}$$

#### 2.2.5. Setting of Threshold Levels

In order to align the selection of the threshold value for spraying with the expected yield loss as described in [28], the regression model is modified from predicting the ratio between weeds and maize to predicting the amount of weed per unit area. The relative expected yield losses chosen to test the MoDiCoVi algorithm were 2.5%, 5%, 20%, 40%, and 80% based on work by [28]. The relative yield loss depends on competitiveness among weed species. However, in this context, this information was ignored, and the expected yield losses were translated into the following threshold values for relative weed coverage: 1.27%, 2.5%, 11.5%, 27%, and 83%.

### 2.3. Evaluating the Experimental Layout

**Figure 5:**
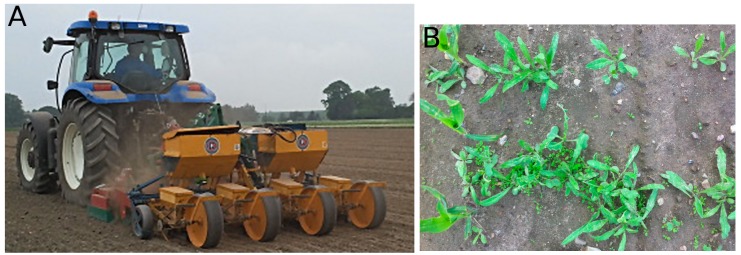
(**A**) 4 June 2013: Seeding the maize while logging the center of the seeding implement; (**B**) 27 June 2013: Unsprayed seeded weeds 23 days after seeding.

The experiment was designed to provide a basis for statistical analysis of the effects of weed control and herbicide usage using different treatment algorithms. There were three experimental variables (on/off spraying and decision treatments (7), seeded/unseeded weeds (2), and camera placements (2)), each with their respective number of variations in parentheses. The desired number of repetition plots (parcels) for each combination was ten, which resulted in a minimum number of 280 plots. Furthermore, two cameras with different short-pass filters were used, each controlling three sprayer valves. This resulted in plots that, in reality, were split between two neighboring 750-mm wide sub-plots. For further details, see the description of the grid-spraying chamber below. The seven instances of on/off spraying treatment decisions were split into two main groups: (1) Control where spraying was always on or always off for the entire plot; (2) MoDiCoVi on/off spraying algorithm with five threshold levels based on weed leaf coverage defined in the previous section. The weed variable defined whether two orthogonal rows of weeds within a plot had been seeded in order to guarantee a minimum weed population beyond what might occur naturally in the field. Two rows of unsprayed seeded weed rows are shown in Figure 5. The camera placement variable determined whether the cameras acquiring images for the MoDiCoVi algorithm were placed above a crop row or between two crop rows. (This was implemented by shifting the route plan for the automated implement carrier by half a row (in width) to the left when placing the camera above the crop rows.) The maize field was located in Flakkebjerg, Denmark (55.326453 N, 11.382436 E). On 4 June 2013, the maize (Zea mays cv. Labriora) crop was seeded with a row distance of 750 mm and a seed spacing of 100 mm, which corresponded to approximately 13 plants per m${}^{2}$. Seeding was carried out with a four-row plot seeder (Kleine FK) mounted on a New Holland tractor (CNH Industrial N.V., London, United Kingdom), with TopCon X20 (TOPCON corporation, Tokyo, Japan) auto-guidance system. The exact center of the seeding tool was logged with an additional 5 Hz Trimble AG-372 RTK GPS (Trimble Inc., Sunnyvale, CA, USA). To ensure the non-uniform distribution of weeds between plots, a mixture of three weed species, Oil-seed Rape (*Brassica napus* L.), Corn Marigold (*Chrysanthemum segetum* L.), and Fat Hen (*Chenopodium album* L.) were sown following the crop seeding in two strips, 200 mm apart and orthogonal to the crop rows for every eight meters corresponding to every second row of plots. The field was divided into 299 plots. Each plot was 3 m × 4 m (oriented along the driving direction). The herbicide MaisTer (containing 300 g·kg${}^{-1}$ a.i. foramsulfuron + 10 g·kg${}^{-1}$ a.i. iodosulfuron + safener 272 g·kg${}^{-1}$ a.i. isoxadifen, Bayer CropScience A/S, Copenhagen, Denmark) was applied to the field with a maximum dosage of 75 g·ha${}^{-1}$ on the first date of application and 150 g·ha${}^{-1}$ on the second. The herbicide was applied on 20 June 2013 and on 2 July 2013, corresponding to 16 and 28 days after seeding, respectively. Seven days after the second herbicide application (35 days after seeding on 11 July 2013) images were captured in a manner similar to that used for the first and second spraying sessions. The images were logged simultaneously along with images from the spraying sessions for the entire area in question, including all areas that were potentially sprayed based on the same images as were used by the spraying algorithm MoDiCoVi and the ground truth.

### 2.4. Automated Trial Execution

A large-scale field experiment for fully automated execution was designed in order to obtain a high degree of consistency in the results. A total of 28 treatment combinations were randomly assigned to the 299 plots, and a fully autonomous robot sprayer performed all treatment combinations in a single pass. The robot sprayer consisted of three major components: (1) An autonomous tool carrier; (2) A grid-spraying implement; and (3) A trailer Hardi ATV spray tank (HARDI INTERNATIONAL A/S, Nørre Alslev, Denmark).

#### 2.4.1. Automated Tool Carrier

**Figure 6:**
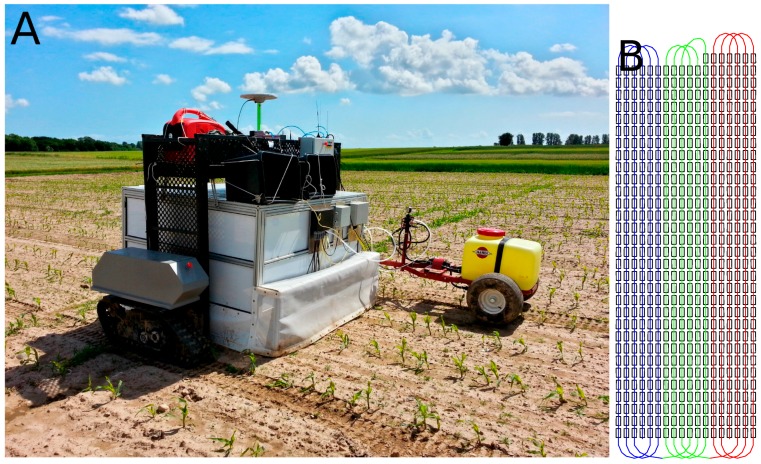
(**A**) Armadillo tool carrier equipped with a vision system and six spray nozzles dragging a Hardi ATV trailer with spray liquid; (**B**) Layout showing all the plots of the trial rotated by 70${}^{\circ }$. The colored lines show the overall route plan passing all plots, and the color changes indicate a pit stop every 900 m to refuel the Hardi ATV trailer carrying the herbicide.

The automated tool carrier, Armadillo IV, was described in [24]. It consisted of two independent track modules mounted on each side of the grid-spraying tool. Each track module contained a 48 V, 5 kW brushless direct current (DC) motor, transmission, and a RoboteQ HBL1650 motor controller. Power was supplied to the module by four 12 V, 65 Ah AGM batteries. The total weight of the track module, excluding the battery case and its contents, was 97 kg. The Armadillo IV robot was controlled by the robot operating system (ROS)-based FroboMind Software Platform described in [29]. Pose estimation was based on input from a VectorNav VN-100 IMU, a Trimble BX982 RTK-GNSS, and hall encoder feedback from the DC motors, which were preprocessed and then used as input to an extended Kalman filter. The Armadillo robot navigated the predefined route plan shown in Figure 6 using an AB line navigator algorithm along crop rows to minimize cross-track errors. Turning at the headland was handled by using pure pursuit. While navigating the route plan, information regarding the entry and exit of plots was continuously communicated by the robot to Linux-based camera computers at the grid-spraying tool. This was handled by a mapping component that loaded a list of polygon coordinates describing the 299 plots. At each position update, the new position was checked against the polygons, and information regarding the current position, velocity, and plot entry/exit ID was forwarded. The set points for the velocity of the Armadillo tool carrier were set to 0.35 m·s${}^{-1}$ for sampling dates 1 and 2, and 0.5 m·s${}^{-1}$ for the final sampling date. The set-point velocities were defined relative to the set image acquisition rate, which in turn was set by the tool. The grid-spraying tool ensured an expected image overlap of approximately 20% and 70% for the first and second set point velocities, respectively.

#### 2.4.2. Grid-Spraying Tool

**Figure 7:**
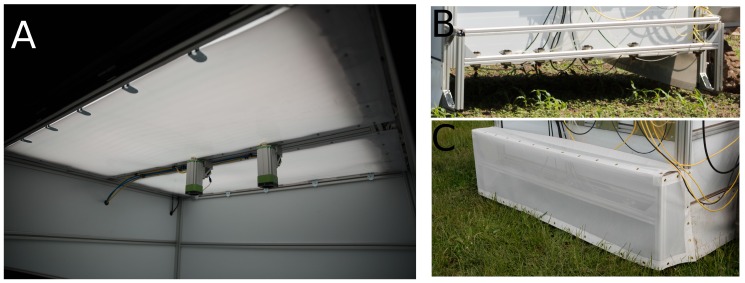
(**A**) The image acquisition chamber with cameras and illumination tubes; (**B**,**C**) Spray chamber with/without windbreaks installed.

The grid-spraying tool consisted of two chambers, one for image acquisition and the other for spraying. The image acquisition chamber (Figure 7) was 1.8 × 1.5 × 1.1 m and was made of alubond with a white (matt) finish shielding against outside illumination, resulting in diffuse reflections on the interior. Inside the acquisition chamber, illumination was provided by 36 Philips TL-D 90 Graphica 58 W fluorescent tubes, providing 5000 K illumination with a color rendition index of 97%. A sheet of translucent polycarbonate was placed in front of the fluorescent tubes (Makrolon GP white) in order to ensure diffuse illumination. This corresponded to an illumination of 78,840 Lumen, which generated an intensity of approximately 30,000 Lux. Inside the chamber, two Basler acA2000-50gc cameras (Basler AG, Ahrensburg, Germany) were mounted with an offset of half a row width (375 mm) relative to the center of the chamber. Furthermore, the cameras were equipped with Azure 1214M5M lenses to facilitate image capture. The cameras were global shutter CMOS cameras with 5.5 μm pixels at a resolution of 2048 × 1088 pixels, each with a 60 dB signal-to-noise ratio (SNR). The focal points of the camera were 920 mm above the ground, and the lenses adjusted to F/4.0, resulting in an airy disc of 5.3 μm. The lenses were focused at 820 mm, resulting in a circle of confusion smaller than 5.5 μm—from 737 mm to 924 mm—producing in-focus images for objects of heights between 4 mm below and 183 mm above the expected soil surface. This corresponded to approximately 2.4 pixels/mm. The Basler cameras had two different IR block filters, one for each camera (the standard Basler camera filter on one; a shortpass filter from Midwest optical systems, model SP700, on the other), between the CMOS imaging sensor and the object. This was performed to test the potential of tailoring the filter for better plant/soil segmentation, as in [30]. The grid-spraying chamber, Figure 7, was a 1800 × 500 × 300 mm chamber mounted with windbreaks to reduce drift. Inside the chamber, six high-speed solenoid Weedseeker VC-01 valves were mounted to turn the spraying on/off. Each valve was mounted with a Hardi medium atomizing LD-01 110 nozzle. The nozzles were mounted 250 mm above the ground with a spacing of 187.5 mm at the boom. The nozzle output was 0.37 L·min${}^{-1}$ at 2.5 bar.

Based on the plot ID and velocity of the robot, the computers determined the treatment combination, especially with respect to the two on/off ground truths and the five threshold levels of the vision-based spraying algorithm. The vehicle velocity was used to set the delay between the image acquisition and potential valve actuation to compensate for the mounting distance between the camera and spraying nozzle. At the same time as the image-based spraying, raw images were stored and geo-tagged for post-data processing and visualization. The frame rates for image acquisition were set to 1 Hz or 4 Hz, depending on whether the laptops were required to run the spraying algorithms in real time or only acquire images, as was the case for the third sampling date. The Hardi ATV spray tank trailed behind the Armadillo tool carrier (Figure 6A). The location of the hinge connecting the trailer significantly limited the robot’s left turning radius, and the route plan thus contained right turns only (Figure 6B). The tank pressure was set to 2.8 bar with the valves closed, which resulted in a pressure of 2.2 bar when all the valves were open.

#### 2.4.3. Data Pre-Processing and Visualization

**Figure 8:**
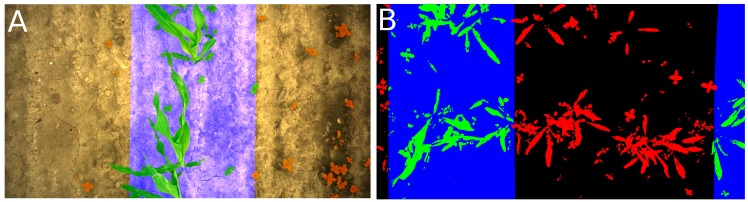
Images from spray session 2 illustrating the inter-row area used to estimate weed coverage. (**A**) The camera is centered above the crop row. The green color represents plant pixels belonging to the estimated crop band (blue), and the red color represents weed pixels belonging to the estimated inter-row area; (**B**) Similar to the left sub-figure except that the camera is located between the two crop rows. Furthermore, the image was acquired at the location of an unsprayed seeded weed strip. The soil is colored black.

The 4 m × 3 m plots used by the automated execution control system were reduced to the width of the six spray nozzles and by half a meter in length at either end of each plot. Hence, the net dimensions of each plot were 3 m × 1.5 m. This was conducted to avoid treatment in areas where the vehicle tracks would be driving. To remove outliers caused by the tool carrier, plots that involved too long or too brief a time interval for driving through them were removed. The plots to be removed were determined in accordance with the amount of time in the driving through: those in the 5% of the upper intervals and 5% in the lower intervals of drive-through times were excluded. This would typically be caused by becoming stuck in mud, a system crash, or similar non-spray/algorithm- related issues. To calculate the reference weed coverage in each plot, logged GPS tracks from the seeder were used to estimate the position of the maize rows. By combining these positions with the known position from the GPS log of the Armadillo robot, locations were projected into the image plane. Based on the projected GPS positions, the path was interpolated using bilinear splines. Morphological dilation was used along the spline to mark the area deemed part of the maize row. The radius of the dilation disc was set to 350 pixels ( 146 mm), thus ensuring that the majority of the crop leaves were covered across all three sampling dates. To enable the leaves of plants to extend beyond the marked area, the centroid of each plant (defined using blob labeling) was used to define whether each plant was considered maize or weed. The weed coverage area was estimated by counting the number of segmented pixels not classified as maize (Figure 8).

MATLAB was used to estimate weed coverage in the images to establish the ground truth. Data was then exported for analysis in R (2012), which was used to pre-process the data and visualize the initial findings. Nozzle activity was estimated based on the recorded spraying log files as the ratio between the on/off nozzle times for the 3-m plots. The estimated weed coverage within a plot was based on the estimated inter-row band shown in Figure 8. The segmented vegetation pixels were counted based on the normalized excess green defined in Section 2.1. The results for nozzle activity and the estimated weed coverage, respectively, were expressed using a standard R box plot grouped by treatment combinations defined by the on/off spraying decision algorithms, the threshold values (inclusive 0% and 100% spraying), on/off seeded weed strips, the camera filter, and the camera position relative to the crop row.

## 3. Results

**Figure 9:**
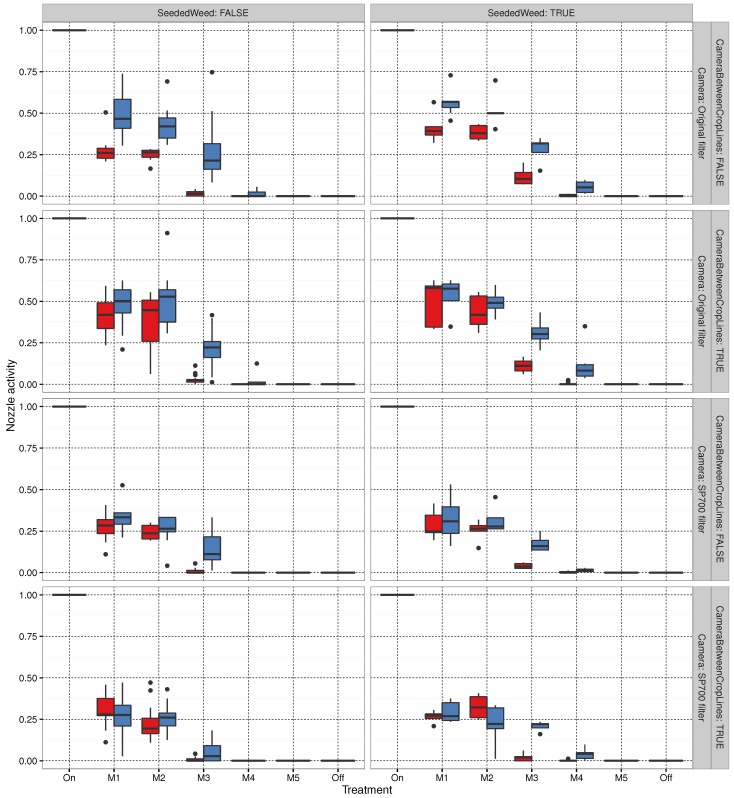
Nozzle activity for each treatment combination. The box plot for the first spraying session is red and the second is blue.

**Figure 10:**
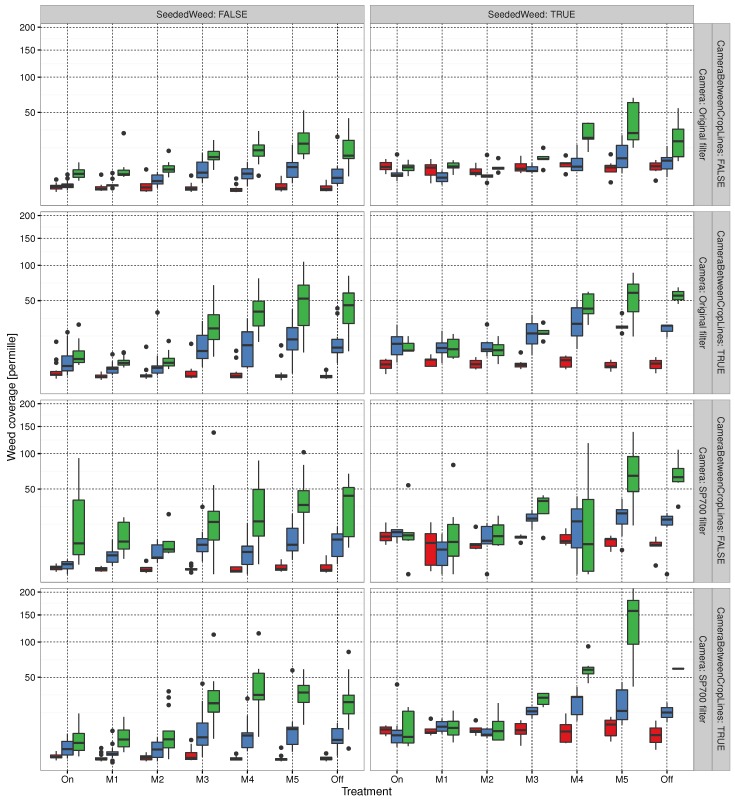
Weed density estimate for each of the three instances of image acquisition in the field. The first scan session is marked in red, the second in blue, and the third in green.

The trial was performed for 299 plots, of which 241 were accepted for further data analysis; the remaining 58 were excluded. This section describes the method of exclusion for the remaining plots and the results from the 241 accepted plots. Pre-processing was performed on the data to exclude invalid instances. A plot was discarded if it did not meet the specifications of the experiment for any of the three sessions. These specifications defined the velocity of Armadillo, and stipulated that the initial area covered by weeds should be between the 0.5% and 99.5% of the data. Armadillo became stuck in muddy conditions due to heavy rain prior to the first day of spraying, resulting in several disturbed plots, which also had to be excluded from the trial. As the spray timing assumed a constant velocity, stopping would cause the spray to be incorrectly positioned. A crude filtration of plots yielding unusually low or high amounts of initial weed coverage at the first spraying session was considered an outlier and permanently excluded from the analysis. The log file from the third session to acquire images contained significantly more extremes with regard to the average time taken to pass a plot. Hence, the 90% quantile used for sessions 1 and 2 was narrowed by removing the additional 5% for the slowest pass times. The number of excluded plots from the trial was within the safety margin for the number of repetitions of the trial. These excluded plots from treatment combinations with only two or three replicates. The impact on treatment replicas should be kept in mind when studying nozzle activity and weed coverage in Figure 9 and Figure 10, respectively. For plots without seeded weeds, the number of replicates was between 7 and 14.

### 3.1. Evaluating Experimental Layout

The seeding of the maize crop went as planned for all plots. In total, 64% of the planned weed strips were seeded because of a limited amount of available seeds. In this trial, weed seeding was carried out to guarantee weed growth in the plots. Prior to the execution of the actual spray procedure, it became clear that natural weed growth in the plots was sufficient even without the seeded weeds. No visual markers or poles were placed in the plot trial. This saved a significant amount of time in setting up the trial. However, when following the Armadillo tool carrier performing automated execution, it was difficult to determine which treatment combination was active at any given time, and to obtain an impression of the performance of the on/off spraying algorithm active at any given time. The route plan included two pit stops after 900 m of driving to enable the refilling of the Hardi ATV trailer. This turned out to be unnecessary because herbicide consumption was lower than anticipated, indicating a measurable reduction.

### 3.2. Automated Trial Execution

By the time the first spraying date arrived, significant efforts had been invested in integrating the Armadillo tool carrier with the grid-spraying tool and its internal sub-components, and calibrating and trimming the dicotyledonous weed quantification algorithm (MoDiCoVi). The day prior had been rainy, and the window of time for the spraying session was restricted to an afternoon on account of forecasted showers the following day. It was therefore decided to use the features derived from training from the previous year, which had the added benefit of rendering overfitting less likely to influence the results. The dataset used was based on work by [26] on another field and growth season, but similar to those in this study. The excess green segmentation threshold values for the two cameras were manually set on the first day of spraying based on images acquired immediately prior to the automated execution. It was noted that the segmentation seemed visually worse for the camera with the standard low-pass filter. While exploring the logged images from the camera with the standard filter, the authors realized that the planned excess green vegetation/soil segmentation would result in a low separability between the two classes despite the artificial and diffuse sources of illumination. This would result in erroneous pruned weed counts compared to the images acquired with the camera using the SP700 filter, which clearly separated vegetation and soil based on excess green. Hence, it was decided to apply statistics-based segmentation using a naïve Bayes classifier, as in [31]. Based on visual evaluation, this significantly improved vegetation identification for the camera that used the standard filter. A relatively simple statistical evaluation was performed to determine whether the two segmentation approaches introduced bias in the vegetation count in the two camera filter selections. The authors could not reject, with 5% confidence, the hypothesis that the different vegetation segmentation methods affected weed coverage estimation (assuming common weed population and coverage for control plots sprayed 0% and 100%).

### 3.3. Sprayer Activity

This sub-section contains plots summarizing the performance of the proposed algorithms. In the field trials, the fraction of time for which the sprayers were active in each plot was logged. Sprayer activity is plotted for the different algorithms in Figure 9. From the figure, it can be seen that, compared with broadcast spraying, the lower thresholds (MoDiCoVi 1–MoDiCoVi 2) of the algorithm yielded usage of approximately 35%, the medium threshold (MoDiCoVi 3) yielded an approximately 5% usage, and the higher MoDiCoVi threshold yielded one of nearly 0%.

For algorithms MoDiCoVi 1 to MoDiCoVi 4, nozzle activity seemed higher for the camera with the standard filter than one with the SP700 filter. With regard to nozzle activity in relation to whether weeds were seeded, there was no clear offset for MoDiCoVi. The area seeded with weeds within a plot covered less than 7% of the area. Hence, it might be difficult to perceive this in the nozzle activity. The camera location seemed not to have had an effect on nozzle activity for the MoDiCoVi algorithm. Nozzle activity seemed higher for the second spraying session, especially in plots with no seeded weeds. In plots with seeded weeds, nozzle activity tended to be identical for MoDiCoVi 1 and MoDiCoVi 2. However, for MoDiCoVi 3 and MoDiCoVi 4, nozzle activity seemed to be highest in the second spraying session. The weed density estimate for each of the runs and threshold levels is shown in Figure 10. It should be noted that, based on visual observations of the control plot sprayed at 100% (on), weeds on the first date were still green on the second spraying date. However, their growth had clearly stagnated. In the third session, seeded weeds had begun to turn yellowish, and some were crumpled. This might have been the cause of the small reduction in weed density between sessions 2 and 3 for the seeded weed plot and the 100% sprayed control plot. The figure shows the common development of weed density estimates for always-spraying (on for the lower MoDiCoVi thresholds (M1 and M2)). Here, the weed counts increased from the first session to the third. Reaching the medium threshold (M3) with a significant rise in weed pixel counts for sessions 2 and 3, and for M4 and M5, there was no clear difference in weed counts compared with not spraying at all.

Based on visual inspection of nozzle activity in Figure 9 and the resulting weed densities in Figure 10, it appears that potential herbicide savings were somewhere between MoDiCoVi 2 and MoDiCoVi 3 if the goal was no significant increase in weed density compared to the 100% sprayed plots. Hence, the potential herbicide savings will be somewhere between 65% and 95% of broad spraying.

## 4. Discussion

The MoDiCoVi algorithm could obtain herbicide savings with no clear increase in weed coverage, which was surprising even when using the camera with poor segmentation. The fact that the authors had trained the algorithm on a dataset from an earlier season indicates that specific training may not be required each time a new field is reached. The authors of [32] showed that weed density was always higher within rows than on inter-row segments unless the latter had been tracked by tractor wheels. This indicated that soil disturbance by the planter and tractor wheels increased weed seed germination and subsequent seedling emergence. Weed cover in undisturbed inter-row areas was generally lower than or equivalent to weed cover in rows. The authors consequently conclude that MoDiCoVi is preferable to simpler methods, which only perform analysis between the crop rows, whereas MoDiCoVi can estimate weed coverage independently of the monocot maize crop leaves in an image. MoDiCoVi does not handle grass weeds at present, which is a weakness of the current version of the algorithm. However, the modification of the MoDiCoVi algorithm to estimate absolute weed coverage nonetheless enables significant potential herbicide savings in the range of at least 75%. The potential savings depend on the growth season and the field in question; however, the obtained savings seem to exist within the results of other research that applies grid-based spraying [7,33]. The potential of MoDiCoVi to handle leaf occlusions seems even better within denser cereal crops, such as winter wheat. However, this has not yet been confirmed. The trial design itself should have been expanded with at least two additional control application treatments, where the plots were only sprayed 100% once, either at the first or second spraying date, in order to evaluate potential herbicide savings. The Armadillo robot added unnecessary complexity to the trial. The grid-spraying tool used a closed illumination chamber excluding all natural light. This ensured strong diffused illumination conditions. This setup was chosen so that the algorithms, and not the segmentation quality, were evaluated. Nevertheless, the segmentation by the camera with the standard filter caused problems. Future work should overcome the need for shutting out natural light. This seems possible considering the image quality obtained in the experiment by [34]. The bispectral system that they used will still be too expensive because the full width of a spraying boom should be sensed in real time. Recent technological developments by [35] in pixel-based spectral coatings, however, have the potential of significant cost reduction, which can facilitate cost-effective multispectral vision systems.

## 5. Conclusions

This study implemented and tested in situ two vision-based algorithms for grid-based on/off spraying. The tests showed that, compared to conventional broadcast spraying, the potential reduction in herbicide usage through the proposed method is 75% while maintaining weed control effects. However, a statistical analysis is needed to confirm this finding. The most promising and robust algorithm in the experiments was the computer-intensive MoDiCoVi algorithm. Additional growth seasons are needed to confirm these findings.

## Figures and Tables

**Figure A1:**
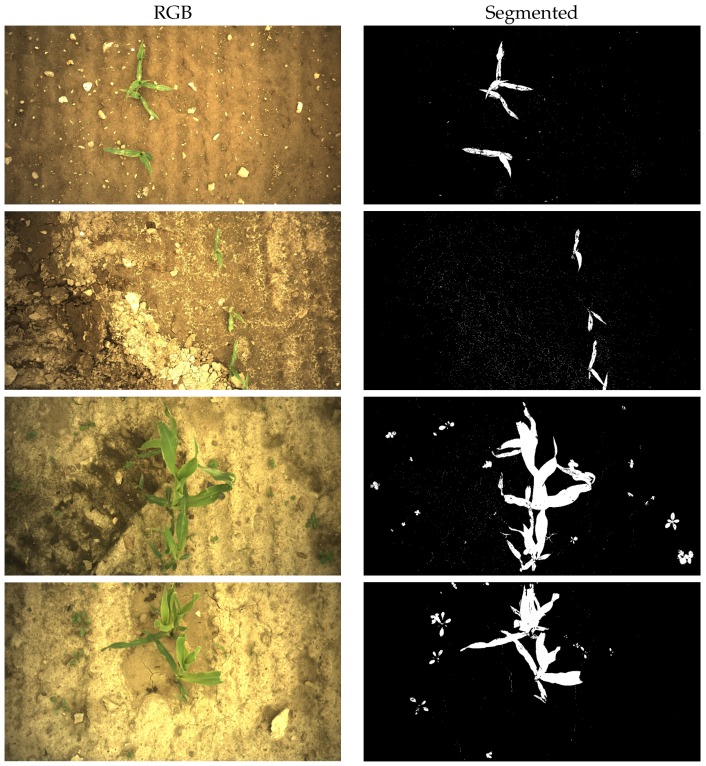

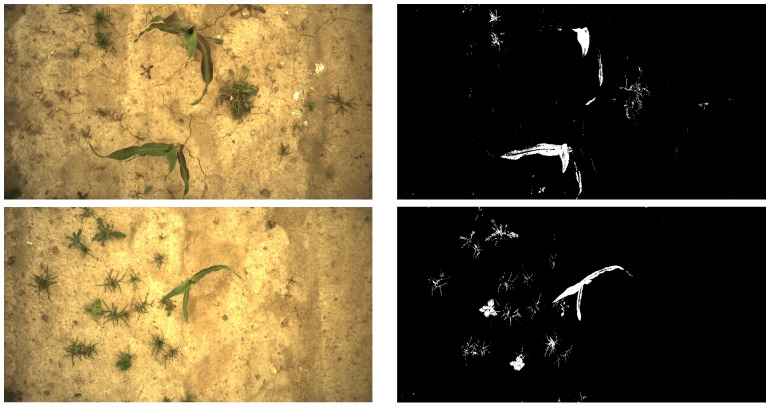
Segmentation examples.

**Figure B1:**
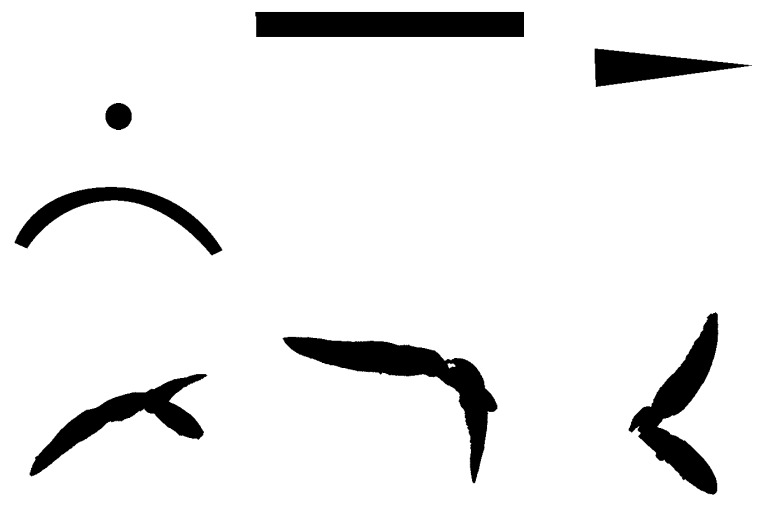

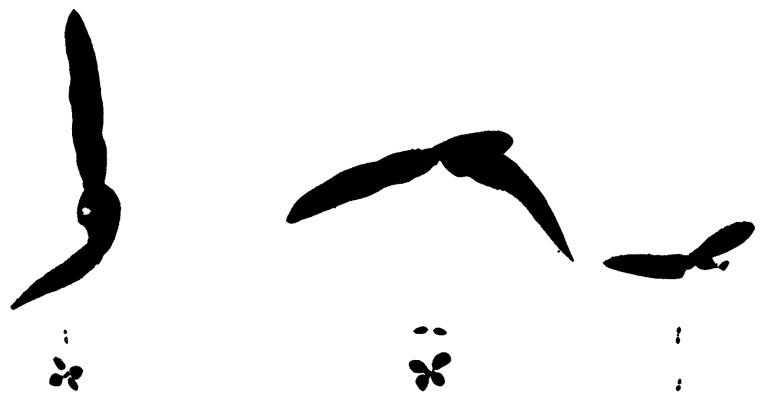
Index of images scaled equally.

**Figure B2:**
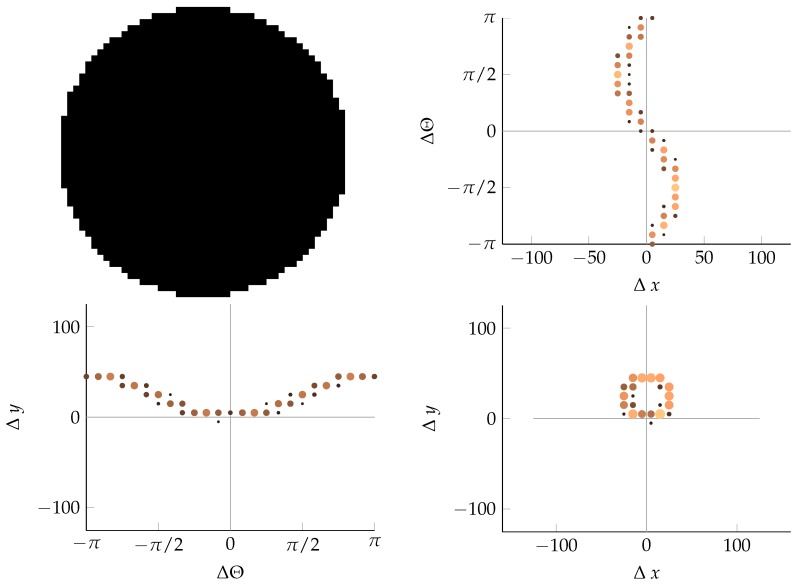
Geometry example circle.

**Figure B3:**
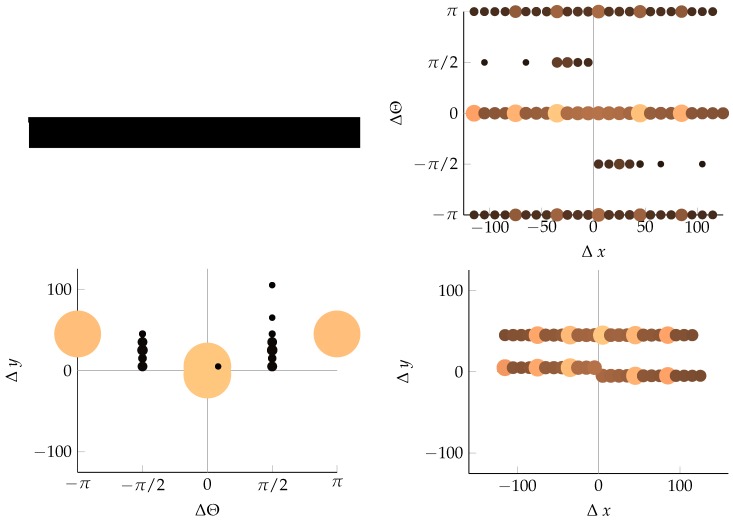
Geometry example rectangle.

**Figure B4:**
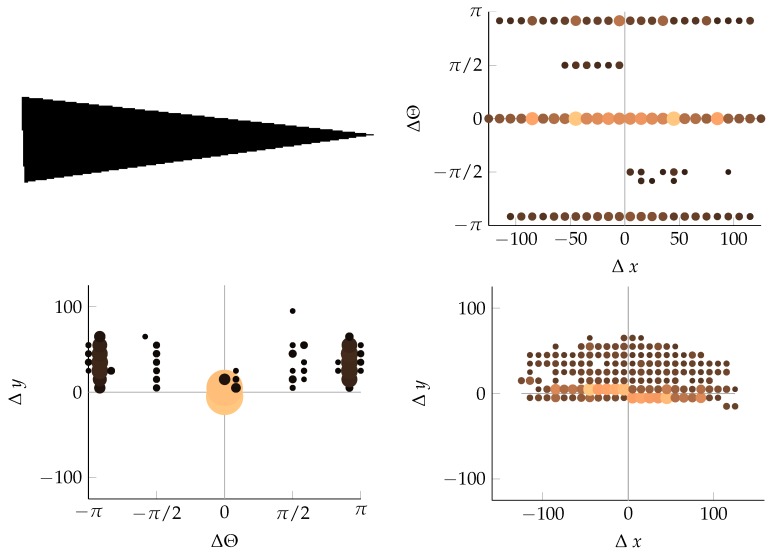
Geometry example triangle.

**Figure B5:**
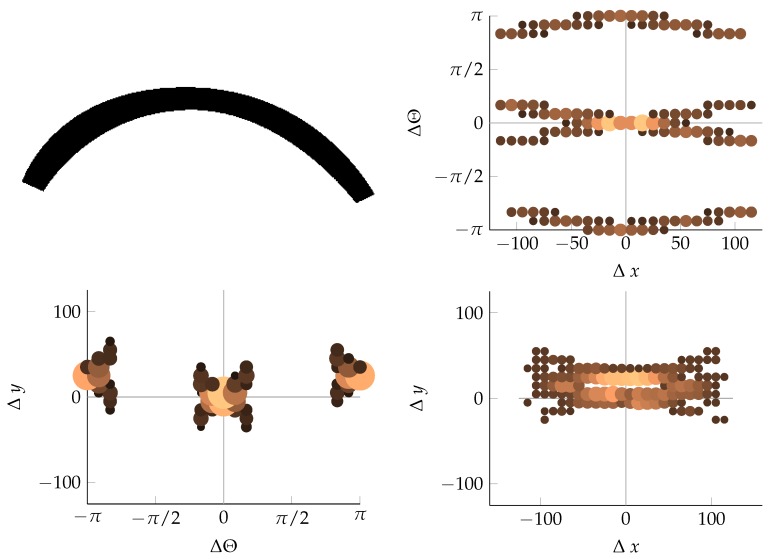
Geometry example arc.

**Figure B6:**
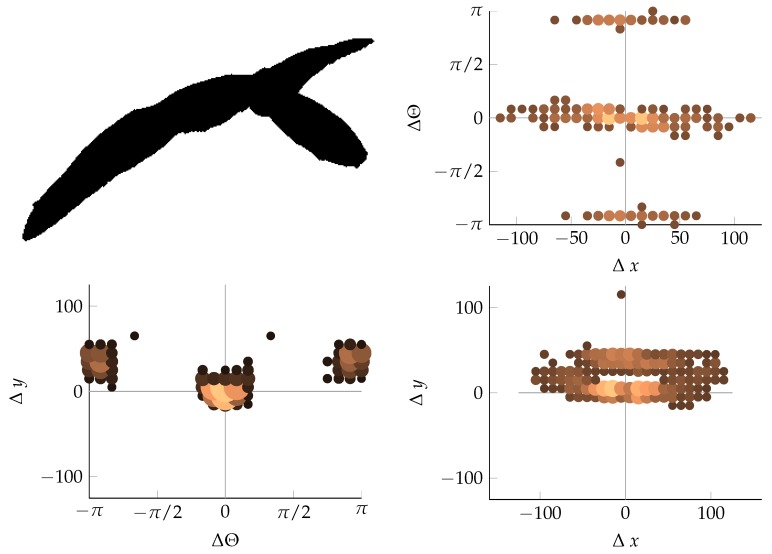
Monocot example 1.

**Figure B7:**
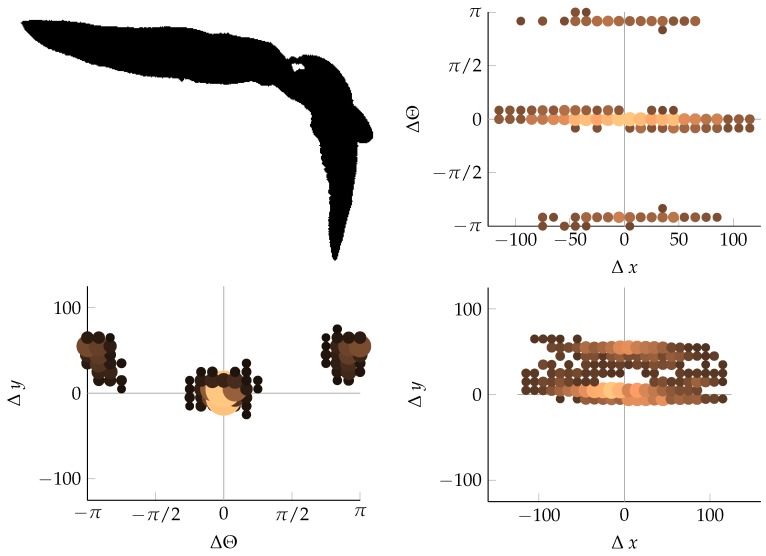
Monocot example 2.

**Figure B8:**
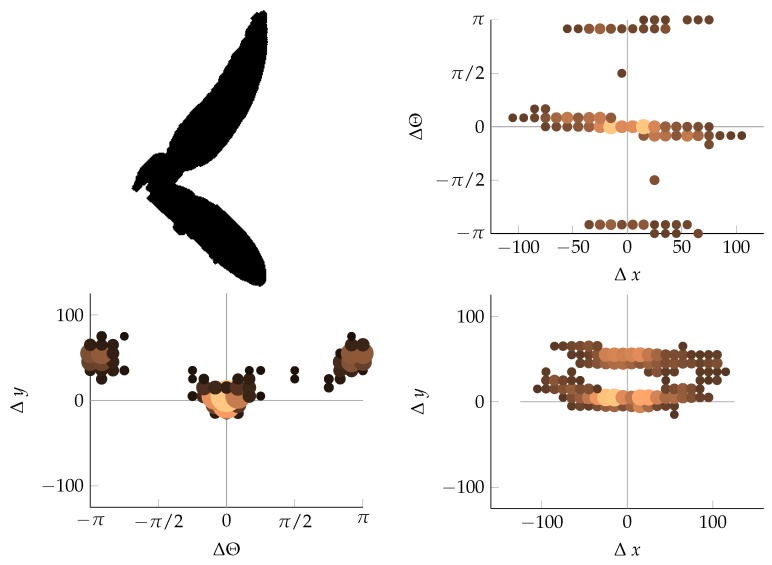
Monocot example 3.

**Figure B9:**
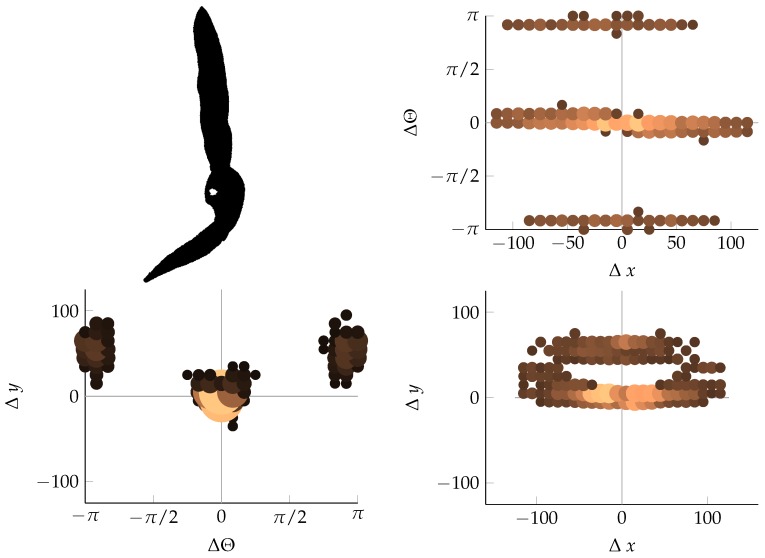
Monocot example 4.

**Figure B10:**
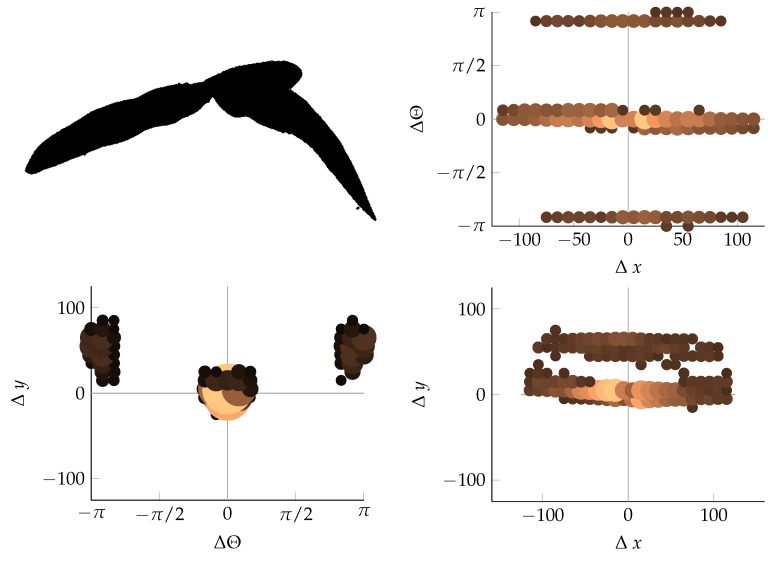
Monocot example 5.

**Figure B11:**
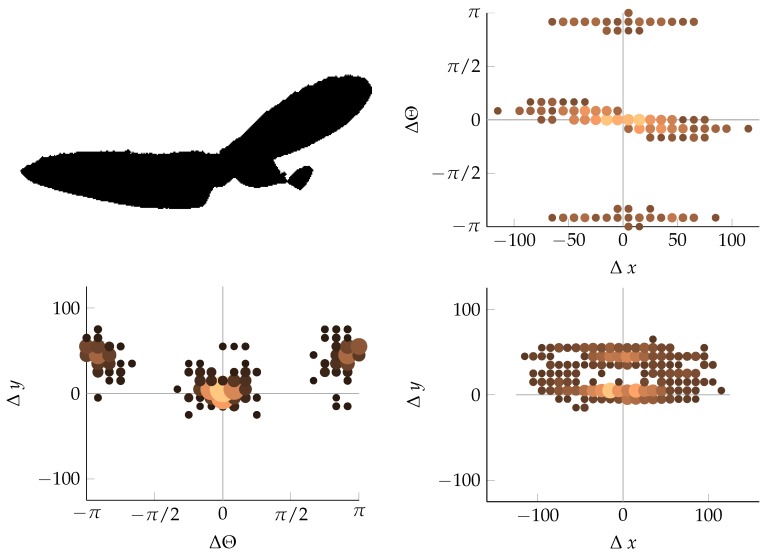
Monocot example 6.

**Figure B12:**
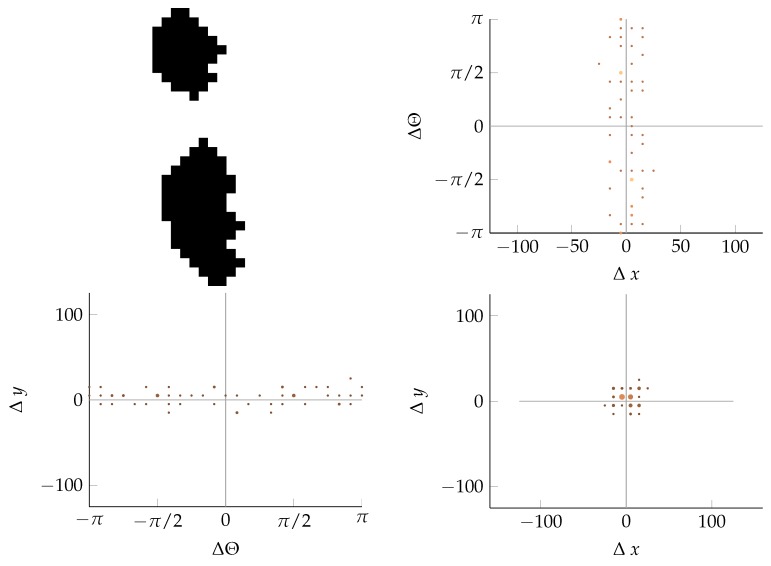
Dicot example 1.

**Figure B13:**
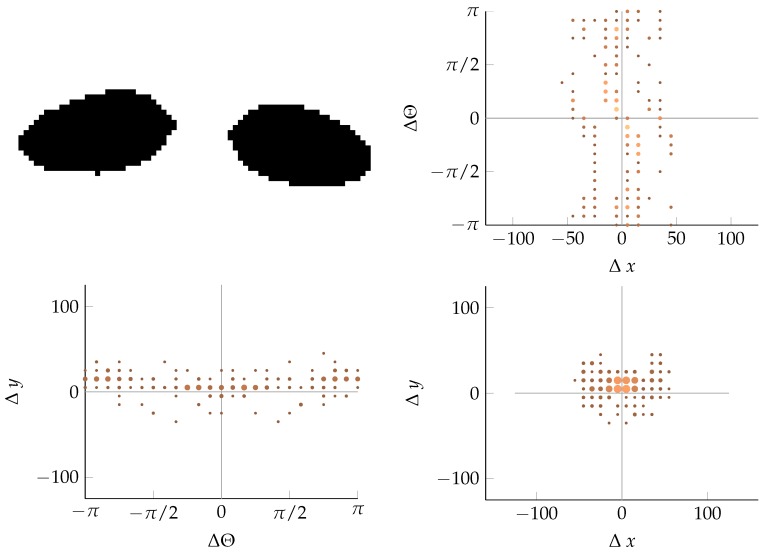
Dicot example 2.

**Figure B14:**
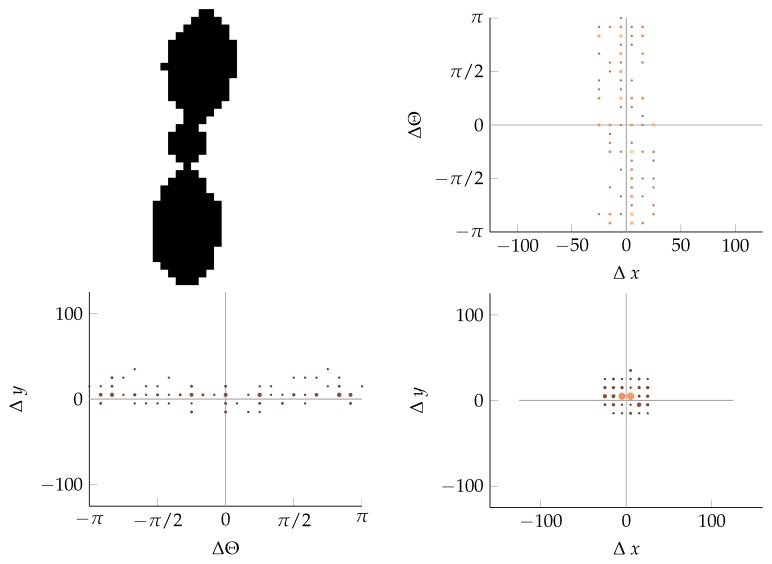
Dicot example 3.

**Figure B15:**
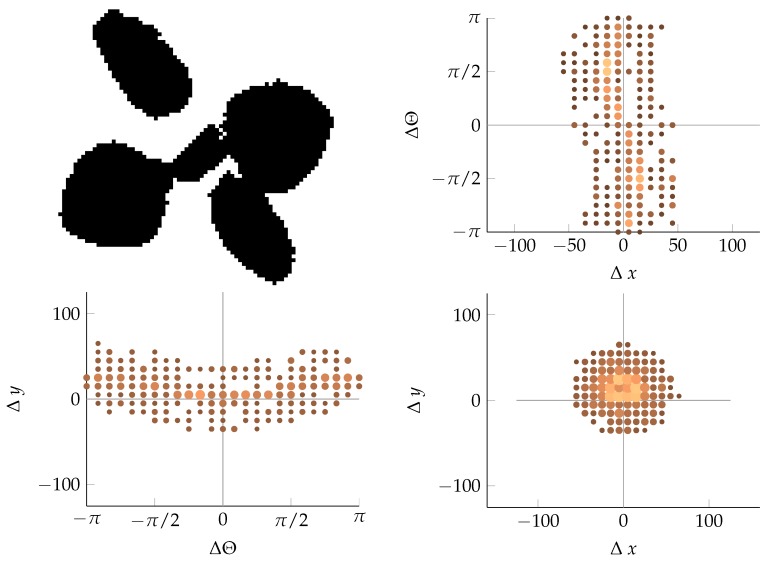
Dicot example 4.

**Figure B16:**
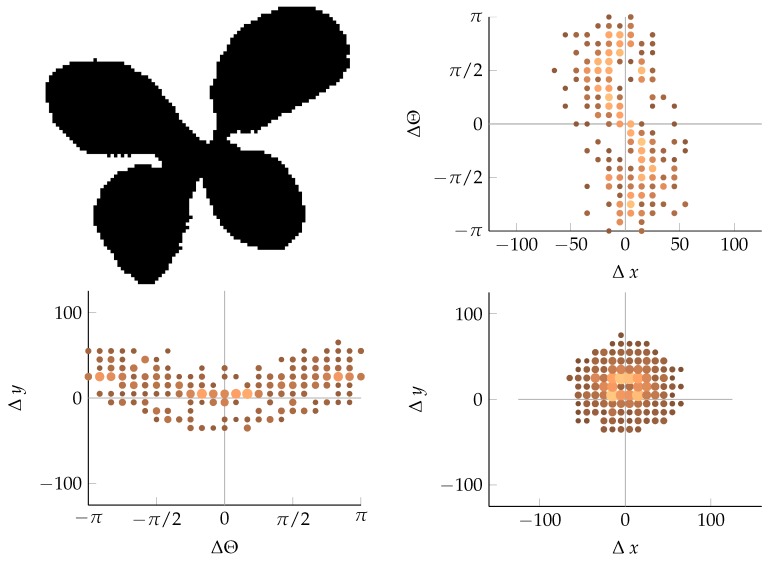
Dicot example 5.

**Figure B17:**
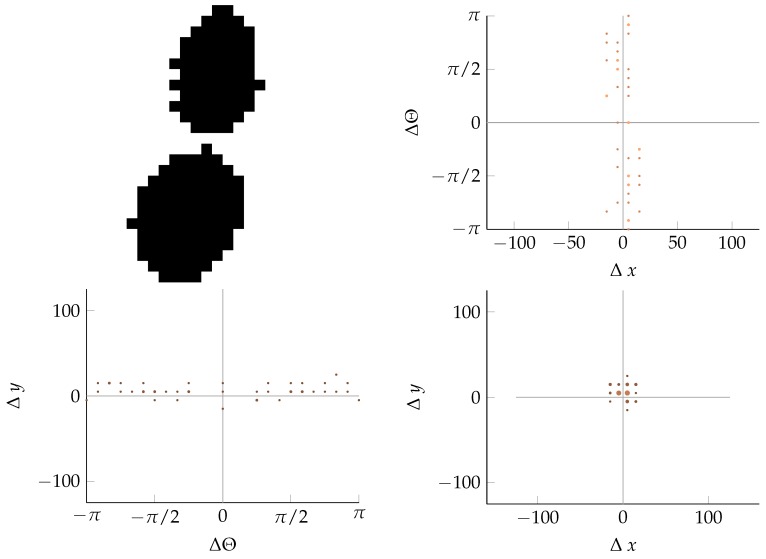
Dicot example 6.

## Appendix B. Example of Shapes as Seen in Relative Coordinates

This appendix shows how each of the shapes shown in Figure B1 appears as interpreted by the internal representation of the MoDiCoVi algorithm. As the images are quite different in size they have been shown here in the same scale, whereas they are scaled to fill the area, when shown together with it’s features. In the following illustrations. First is shown a set of general geometric shapes, then a set of monocotyledons and last a set of dicotyledon examples. The order in which they apear in Figure B1 is the same order as in which they are shown in the illustrations.
